# Influence of different restorative materials on the stress distribution in dental implants

**DOI:** 10.4317/jced.54554

**Published:** 2018-05-01

**Authors:** Carlos-Eduardo Datte, João-Paulo-Mendes Tribst, Amanda-Maria-de Oliveira Dal Piva, Renato-Sussumu Nishioka, Marco-Antonio Bottino, Alexandre-Duarte M. Evangelhista, Fabrício M de M. Monteiro, Alexandre-Luiz-Souto Borges

**Affiliations:** 1 DDs, MSc, PhD student at Department of Dental Materials and Prosthodontics, São Paulo State University (Unesp), Institute of Science and Technology, São José dos Campos, SP, Brazil. Avenida Engenheiro Francisco José Longo, Jardim São Dimas, São José dos Campos, SP, Brazil; 2 DDS, MS, PhD, Professor at Department of Dental Materials and Prosthodontics, São Paulo State University (Unesp), Institute of Science and Technology, São José dos Campos, SP, Brazil. Avenida Engenheiro Francisco José Longo, Jardim São Dimas, São José dos Campos, SP, Brazil; 3 Private Pratice at Instituto de Odontologia São Paulo (IOSP), São José dos Campos, SP, Brazil. Av Pensilvânia, Jardim Florida, Jacareí , SP, Brazil; 4 DDs, MSc student at Department of Dental Materials and Prosthodontics, São Paulo State University (Unesp), Institute of Science and Technology, São José dos Campos, SP, Brazil. Avenida Engenheiro Francisco José Longo, Jardim São Dimas, São José dos Campos, SP, Brazil

## Abstract

**Background:**

To assist clinicians in deciding the most suitable restorative materials to be used in the crowns and abutment in implant rehabilitation.

**Material and Methods:**

For finite element analysis (FEA), a regular morse taper implant was created using a computer aided design software. The implant was inserted at the bone model with 3 mm of exposed threads. An anatomic prosthesis representing a first maxillary molar was modeled and cemented on the solid abutment. Considering the crown material (zirconia, chromium-cobalt, lithium disilicate and hybrid ceramic) and abutment (Titanium and zirconia), the geometries were multiplied, totaling eight groups. In order to perform the static analysis, the contacts were considered bonded and each material was assigned as isotropic. An axial load (200 N) was applied on the crown and fixation occurred on the base of the bone. Results using Von-Mises criteria and micro strain values were obtained. A sample identical to the CAD model was made for the Strain Gauge (SG) analysis; four SGs were bonded around the implant to obtain micro strain results in bone tissue.

**Results:**

FEA results were 3.83% lower than SG. According to the crown material, it is possible to note that the increase of elastic modulus reduces the stress concentration in all system without difference for bone.

**Conclusions:**

Crown materials with high elastic modulus are able to decrease the stress values in the abutments while concentrates the stress in its structure. Zirconia abutments tend to concentrate more stress throughout the prosthetic system and may be more susceptible to mechanical problems than titanium.

** Key words:**Finite element analysis, dental implants, ceramic.

## Introduction

Considering that dental implants present high success rates in the rehabilitation of aesthetic and functional restorations in patients with partial or total dental loss, failures in osseointegration could occur after prosthesis installation (1-3). The use of morse taper implants with 1-2 mm below the alveolar bone crest favors the maintenance of peri-implant tissue (4). When associated with platform switching abutments and masticatory loads, this implant promotes favorable stress dissipation in bone tissue (5,6) with less microstrain in the cervical region (7); it also improves biological sealing with reduction in bone loss (8-10).

The most important reason to investigate the stress distribution in abutments and microstrain in crestal bone around implants is the possibility to provide sufficient information for implant planning, optimizing the implant installation in areas with different bone characteristics (11). In spite of this, masticatory overload is one of the primary factors for fractures and dental implant loss (12).

When the clinic uses prosthetic pieces in materials with different elastic modulus, these components can generate different stress and strain in the implant and peri-implant bone (4,13,14). For abutments the most common material used is titanium. Titanium’s reliable mechanical behavior and biocompatibility is well-documented in literature (15). Titanium abutments can be defined as the gold standard for implant rehabilitations. An alternative to titanium are zirconia abutments. They present similar survival and improved aesthetics in peri-implant tissue (16). Zirconia partially stabilized by yttrium (YTZP) has excellent mechanical properties (17) such as hardness (1200 HV), corrosion resistance, elastic modulus (210 MPa), flexural strength (900-1200 MPa), compression resistance (2000 MPa), toughness (7-10 MPa), biocompatibility, good soft tissue stabilization and low plaque retention (18-20).

Another important decision for clinicians is the choice of crown material. A large variety of indirect materials exists to manufacture the restorations. Ceramic materials have become a commonly used material for dental prosthesis because it presents aesthetic and long-term resistance (20). Also, the technique of using ceramic crowns onto implants has been proven successfully in the long term (1-3). Considering the rigidity of these materials, the elastic modulus can vary between the modulus of a zirconia to the elastic modulus of a hybrid ceramic with high resilience (21) due to its polymeric matrix.

Regarding all combinations between abutment and ceramic crown, it is not clear to the clinician which one is the best treatment option to dissipate the generated stresses, and thus to ensure greater longevity. The aim of this study was to assist the clinicians in deciding the most suitable restorative materials to be used in implant rehabilitation in the posterior regions. The lower generation of strain in bone tissue and stress in the abutment/implant set were the guides for this choice aiming for treatment longevity.

## Material and Methods

-Tridimensional model

A regular morse taper internal connection implant (DriveCM Acqua, Neodent, Curitiba, Paraná, Brazil) was created according to the manufacturer’s dimensions (4.3 x 10 mm), using CAD (Computer Aided Design) software (Rhinoceros 5.0, SR8, McNeel North America, Seattle, WA, USA). Next, the model received an anatomic prosthetic solid abutment. The implant was inserted at the center of a three-dimensional bone model (40 x 40 x 20 mm) with 3 mm of exposed threads. An anatomic cemented prosthesis representing a first upper molar was modeled and placed on the abutment. The mechanical properties of polyurethane were used to simulate bone structure.

-FEA processing

After modeling, the 3D model was imported to analysis software (ANSYS 17.0, ANSYS Inc., Houston, TX, USA). Each material was then assigned as homogeneous, linear and isotropic to perform the static analysis. The information of Young’s modulus and Poisson’s ratio were selected from the literature ( Table 1) (22-26). To simulate absence of joint defects, and all contacts were considered bonded. Group division occurred based on a combination between the crown (4 levels) and abutment (2 levels) material, totaling 8 groups.

**Table 1:**
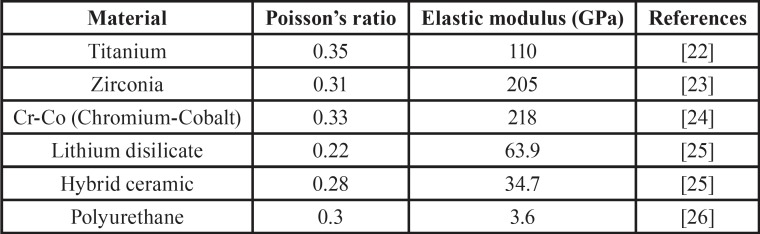
Material properties used to perform the static analysis.

-Mesh generation

The complex geometry was automatically subdivided in tetrahedral elements that formed the mesh. The number of finite elements was 452,561 with 724,131 nodes. These parameters were achieved with a mesh convergence test (10%) to guarantee that the mesh could not interfere in the results (26).

-Loading and fixations

The center of the crown was defined as the loading area according to the defined area in CAD software. An axial load (200 N) was applied in Z axis direction (Apical). The base of the polyurethane block was selected for the system fixation, ensuring movement restriction (26).

-Required results

The results were required according to the failure of ductile solids (27), following Von-Mises criteria. For the peri-implant tissue, the required result was in micro strain based on previous studies that defined these results as important to prevent bone reabsorption (26,27). Any component of the system that presents results with a difference in stress peaks between the groups greater than 10% will be defined as significant (27).

-*In Vitro* Strain Gauge

**Figure 1:**
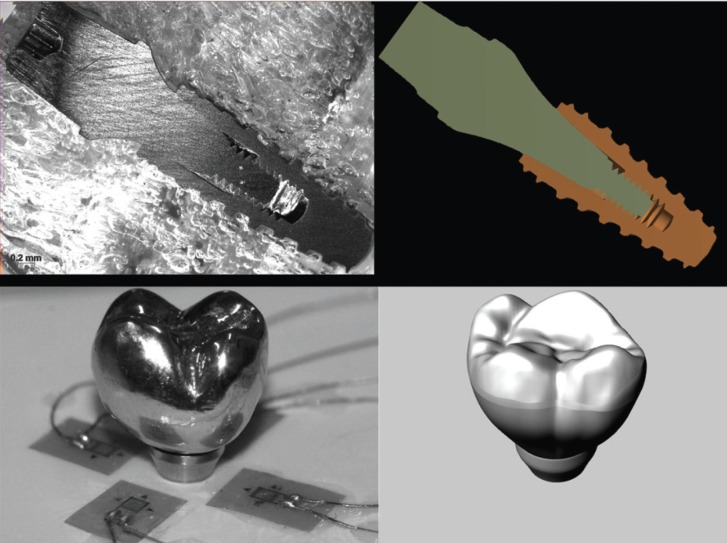
Experimental sample and three-dimensional model with similar geometries.

For the in vitro analysis, an identical sample was made following the same characteristics of the 3D model (Fig. 1); four strain gauges (L2A-06-062LW-120; Vishay, Raleigh, NC, USA) were placed on the surface (polyurethane block) around the implant in locations where relatively large strain values were determined by FEA. The alignment of the gauges was in the direction of maximum strain and parallel to the X or Z axis and perpendicular to the Y axis. Each strain gauge was connected separately, and the four strain gauges were arranged in series to form a one-fourth Wheatstone’s bridge. The wires from the strain gauges were connected to a multichannel bridge amplifier to form one leg of the bridge. A computer (Intel 775P Pentium 4 Q6600; Acer, Miami, FL, USA) was interfaced with the bridge amplifier to record the output signal of the polyurethane surface. Data acquisition system software (System 5000 Model 5100B; Vishay) was used to record the data (27).

-Verification of 3D Model and results validation

**Figure 2:**
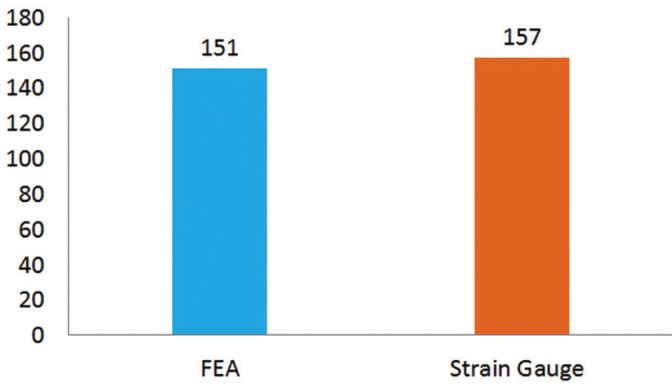
Bar Graph of strain mean calculated with FEA and strain gauge in cervical region of dental implant.

The selected crown to perform a direct comparison between mathematical (FEA) and experimental (Strain gauge) were the Cr-Co crown due to the facility in manufacturing this material and simplicity for polishing. As the model was identical for the 8 groups and only the elastic modulus changed between them, just one crown needed to be evaluated. The load applied imitates the loading during FEA, in the center of the crown with 200 N on the universal testing machine (DL-1000; Emic, São José dos Pinhais, PR, Brazil). The magnitude of micro-strain was recorded in µm/µm. This procedure was made in triplicate. The mean strain around the implant was calculated and plotted on a bar graph (Fig. 2) according to both the methodologies used in this research to show the consistency between the in vitro and FEA results. The mean of FEA results were 3.83% lower than strain gauge mean of measurements.

## Results

**Figure 3:**
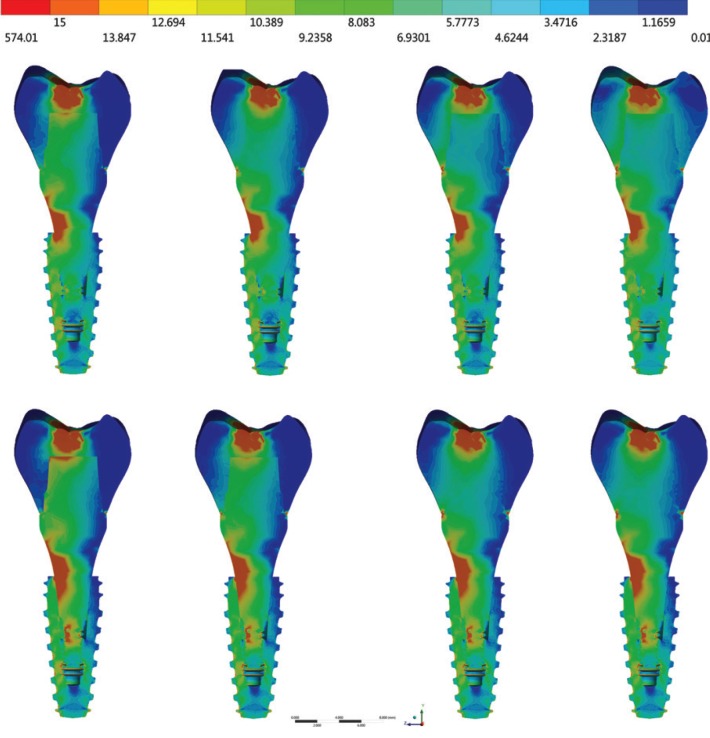
Von-Mises stress in set. In the first row: groups with titanium abutments; and in the second row: groups with zirconia abutments. From left to right: crowns of hybrid ceramic, lithium disilicate, chrome cobalt and zirconia.

**Figure 4:**
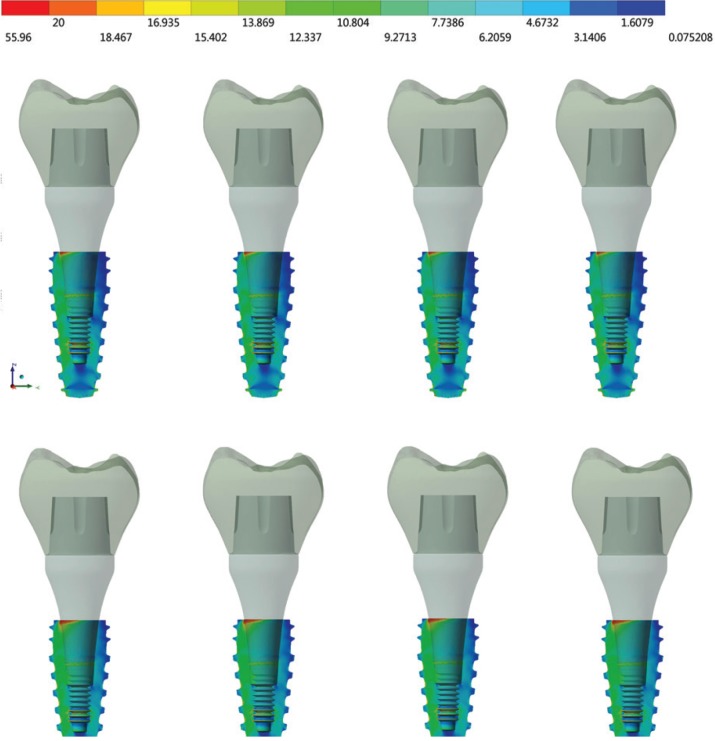
Sagittal view of Von-Mises stress in the dental implant. In the first row: groups with titanium abutments; and in the second row: groups with zirconia abutments. From left to right: crowns of hybrid ceramic, lithium disilicate, chrome cobalt and zirconia.

**Figure 5:**
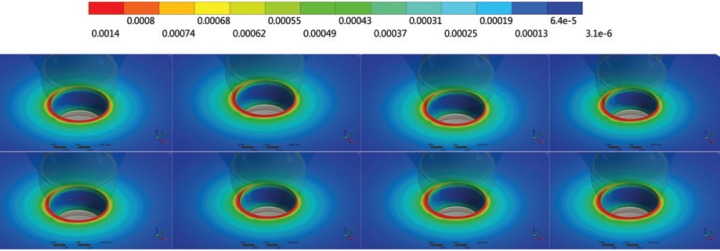
Perspective view of micro strain in peri-implant tissue. In the first row: groups with titanium abutments; and in the second row: groups with zirconia abutments. From left to right: crowns of hybrid ceramic, lithium disilicate, chrome cobalt and zirconia.

**Figure 6:**
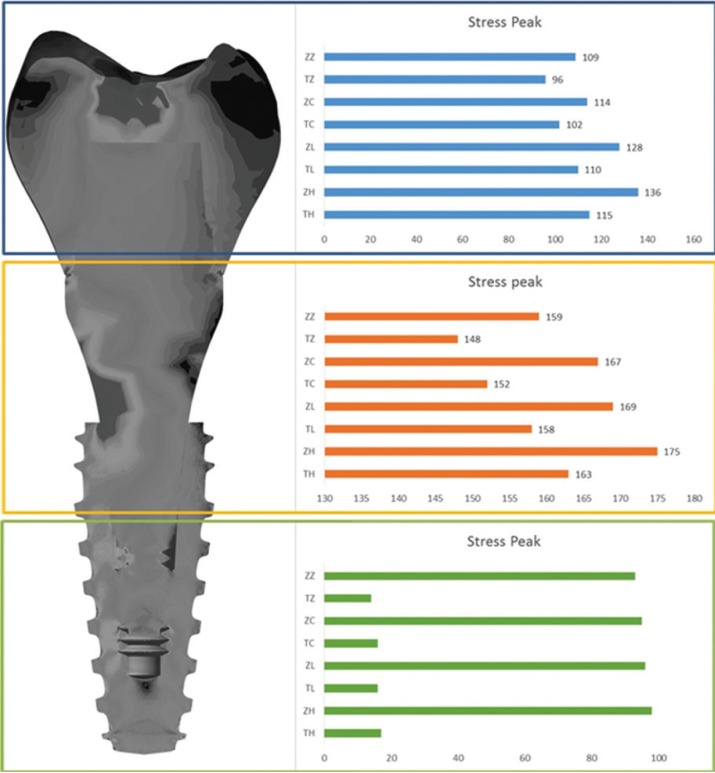
Stress peak in three different regions of the set: Crown region, Implant’s cervical and apical region, according to the groups: ZZ – zirconia abutment with zirconia crown; TZ – titanium abutment with zirconia crown; ZC – zirconia abutment with Cr-Co crown; TC – titanium abutment with Cr-Co crown; ZL – zirconia abutment with lithium disilicate crown; TL – titanium abutment with lithium disilicate crown; ZH – zirconia abutment with hybrid ceramic crown and TH – titanium abutment with hybrid ceramic crown.

For Von-Mises stress generated in each group, a qualitative view allows for perceiving an increase in stress with zirconia abutments when compared to titanium ones (Fig. 3). According to the crown material, it is possible to note that the increase of elastic modulus reduces the stress concentration in all system (abutment/implant/bone). With a sagittal view of the implant (Fig. 4), it is possible note that there is no significant difference (10%) between the groups from the internal threads of implants. No difference was reported between the groups (10%) for the bone micro strain (Fig. 5). The set of restoration (crown, abutment and implant) was divided into three distinct parts and the stress peak of each region was calculated to allow a quantitative comparison between the groups using bar graphs (Fig. 6). The combination of hybrid ceramic crown with zirconia abutment presents the worst biomechanical behavior in the crown region and cervical region. For the apical region of the set, the factor “crown material” was not significant and the factor “abutment material’ showed a very increased magnitude in stress peaks for all groups with zirconia abutments.

Both implemented methodologies do not require statistical analysis because FEA consists of a mathematical method with absolute values. In addition, Strain Gauge was used to validate the computational models with the in vitro test using one sample per group. According to the similarity (3.83%) of results from FEA and Strain Gauge (Fig. 2), it was possible to observe that the models were considered valid and showing that conclusions only from FEA methods are possible.

## Discussion

Proper treatment planning and a sound understanding of restorative aspects of dental implants can prevent most implant failures (8,27). Although the bone remodeling process is constantly dependent on the masticatory load (28), the overload (29) may cause damage to the alveolar bone, thereby promoting loss of osseointegration (30).

The choice of a restorative material is an important decision because it could influence cases of excessive biting force or parafunctional habits. It could also prevent bone tissue from damage due to the fact that bone behavior depends on load magnitude (28). However, it could also be found in the literature that restorative material has no influence on the implant’s survival (1). In the evaluated situation with a morse taper implant, the restorative material seems to have no influence on bone strain. This result favors the clinician who is concerned with the longevity of the treatment, and who can then associate this implant with an aesthetic material. Moreover, the use of zirconia abutments that provide better aesthetics was also not harmful to bone tissue, making it feasible to be used when there is the concern of osseointegration longevity (Fig. 5). These results corroborate with authors who did not find mechanical damage to the bone tissue when using zirconia on implants (29).

The use of an abutment made in zirconia is justified because it is a biocompatible material suitable for maintaining the health of hard and soft tissues (19). Zirconia biocompatibility seems to be more favorable in the perimplant region due to better fiber insertion than titanium (31,32). Despite this, the results in stress suggest that zirconia abutments should be used with caution, since the main region of stress concentration were the abutment’s threads (Fig. 3). This may be associated with fracture reports (33) as the maintenance of torque does not appear to be affected (34). No stress was suggested to be a strain promoted by the abutment in the internal threads of the implant (Fig. 4). However, it is worth emphasizing that if the abutment is released due to the concentrated stress in its threads or even fracture, the system biomechanics will be altered and the implant will have unexpected behavior. Considering the crown material, only the regions of the crown itself and the cervical region of the set were influenced by the different elastic modulus (Fig. 6).

The crown in a material with less elastic modulus concentrated less stress on its structure, which more easily stressed the interface with the abutment and the region above the prosthetic connection. Such results are supported by similar findings in previous studies (21,35). This increase in magnitude suggests probability of initial fracture in these regions of stress concentration, in addition to the possibility of debonding due to the heterogeneous passage of stress between abutment and crown. As the elastic modulus of the crown increased, all previously described effects decreased.

Although the evaluated crowns have different fracture strength profiles and even survival due to their microstructure (21), the choice of restorative material can decrease the stress concentration at the interface with the abutment. For example, when the elastic modulus of the crown approximates the elastic modulus of the abutment as in the case of the crown and abutment in zirconia or crown in Cr-Co and abutment in titanium. In this way, materials with lower elastic modulus could have better mechanical behavior if they were used with similar abutments, such as hybrid abutments made with perforated ceramic blocks.

The limitations of this study consist in using homogeneous geometries which means an absence of internal defects, and no simulation of factors present in the oral cavity such as temperature, pH variation or patient’s hygiene (29). These limitations do not invalidate the results, but suggest that they should be carefully evaluated and used to complement clinical experience in correlation with other papers. From the obtained results it was possible to validate the 3D model and conclude that:

1. Restorative materials used in the manufacture of monolithic crowns on unitary morse taper implants are not capable of influencing bone strain;

2. Zirconia abutments tend to concentrate more stress throughout the prosthetic system and may be more susceptible to mechanical problems than titanium abutments;

3. The crowns with high elastic modulus are able to decrease the stress values in the abutments, while the crowns with low elastic modulus decrease the stress in the crown.
